# Detecting cell lysis using viscosity monitoring in *E. coli* fermentation to prevent product loss

**DOI:** 10.1002/btpr.2292

**Published:** 2016-05-17

**Authors:** Joseph M. Newton, Desmond Schofield, Joanna Vlahopoulou, Yuhong Zhou

**Affiliations:** ^1^ Dept. of Biochemical EngineeringUniversity College LondonGower StreetLondonWC1E 6BTU.K.; ^2^ Research & Development, Procellia Ltd, Netpark Incubator, Thomas Wright WaySedgefieldCounty DurhamTS21 3FDU.K.

**Keywords:** fermentation process monitoring, E. coli, viscosity, cell lysis, product leakage

## Abstract

Monitoring the physical or chemical properties of cell broths to infer cell status is often challenging due to the complex nature of the broth. Key factors indicative of cell status include cell density, cell viability, product leakage, and DNA release to the fermentation broth. The rapid and accurate prediction of cell status for hosts with intracellular protein products can minimise product loss due to leakage at the onset of cell lysis in fermentation. This article reports the rheological examination of an industrially relevant E. coli fermentation producing antibody fragments (Fab'). Viscosity monitoring showed an increase in viscosity during the exponential phase in relation to the cell density increase, a relatively flat profile in the stationary phase, followed by a rapid increase which correlated well with product loss, DNA release and loss of cell viability. This phenomenon was observed over several fermentations that a 25% increase in broth viscosity (using induction‐point viscosity as a reference) indicated 10% product loss. Our results suggest that viscosity can accurately detect cell lysis and product leakage in postinduction cell cultures, and can identify cell lysis earlier than several other common fermentation monitoring techniques. This work demonstrates the utility of rapidly monitoring the physical properties of fermentation broths, and that viscosity monitoring has the potential to be a tool for process development to determine the optimal harvest time and minimise product loss. © 2016 The Authors. Biotechnology Progress published by Wiley Periodicals, Inc. on behalf of American Institute of Chemical Engineers, 32:1069–1076, 2016

## Introduction

Bacterial cell death and lysis is often observed in the late stage of the fermentation process. Understanding this phenomenon has significance both scientifically and commercially. Traditionally, cell lysis is considered a consequence of “unbalanced growth” at the end stage of the bacterial life cycle. However, despite the numerous studies on autolysis, our knowledge is still limited. Lysis phenomena in bacterial fermentations may be influenced by a variety of different factors including environmental conditions in the fermenter such as shear stress or poor mass and oxygen transfer, toxic waste product build‐up, metabolic burden from excessive recombinant protein expression, as well as internal stresses from a build‐up of product in the periplasm.

Antibody fragments that exhibit antigen‐binding properties are a relatively new class of therapeutics entering the market.^1^ *E. coli* is heavily used as a microbial host to express these recombinant proteins as glycosylation is not needed,^2^ it has well‐characterized genetic properties and can be grown using inexpensive media.^3^ Advances in cell culture technology have also led to high cell density fermentations, which not only significantly increases product titre but also increases complications with respect to mass transfer in the dense population.^4^, ^5^, ^6^

*E. coli* produce Fab' fragments that can be routinely targeted to the periplasmic space, a concentrated gel‐like matrix in the space between the inner cytoplasmic membrane and the bacterial cell wall.^3^ However, the capacity of the periplasm is limited, for example, Fab' fragments will leak when exceeding 6% of the volume of the periplasm (data not shown). During fermentation, as the limit of the periplasm is reached, cells begin to lose viability and leak the Fab' product and other intracellular content to the fermentation broth. Apart from product loss in late stage fermentation, the remaining viable cells become more fragile and break fourfold more than those harvested at an earlier stage, if subjected to the equivalent shear level of that in an industrial centrifuge.^7^, ^8^, ^9^, ^10^ As cell lysis occurs in late stage fermentation, leakage of product to the fermentation broth also acts to increase the broth viscosity, as large quantities of chromosomal DNA and other intracellular content are released simultaneously into the broth.^9^, ^11^ High viscosity broths cause additional complications downstream. For example, clarification efficiency in centrifugation is inversely dependent on the viscosity^12^ and studies have previously been carried out investigating the effects of bacterial cell culture age on microfiltration performance,^13^, ^14^ showing that release of intracellular material (such as DNA) due to cell death was found to increase fouling and resistance, hence reducing performance.

Monitoring lysis is particularly important for processes with host cells that store the product in the periplasmic space, as product loss due to leakage occurs at the point of lysis. However, it is difficult to observe directly in fermentation, because it is inherently complex and current analytical technologies are unable to rapidly and accurately monitor the shift between optimal intracellular product concentration and leakage to the fermentation broth. Current industrial processes focus on solving this problem by monitoring cell density, product titres, leakage and cell viability to determine harvesting time.^8^, ^15^ Optical density (OD_600_) is used to monitor biomass growth in fermentation. However, OD measurements only give an indication of total biomass obscuring the light path, and provide no insight into viable biomass.^16^ This means that OD measurements systematically underestimate lysis in late stage fermentation and miss the critical point of product leakage. Viable biomass can also be monitored online using capacitance probes, which use dielectric spectroscopy to measure the electrical capacitance of the cell suspension at various radio frequencies. However, capacitance measurements tend to perform poorly in late stage fermentation, often missing the onset of cell lysis (nonviable cells will still hold a certain amount of charge and exhibit some form of capacitance).^17^, ^18^

Flow cytometry is regularly employed to monitor cell viability in fermentation; however, it requires a lengthy and complicated staining procedure that also requires postmeasurement data analysis. All cell‐based monitoring techniques can only detect existing cells and are unable to measure the lysed cells, making it infeasible to monitor lysis effectively. However, the intracellular content from lysed cells will still remain in the bioreactor. Thus, measuring the protein or DNA leakage can give an indication of cell lysis. HPLC is commonly used to monitor product leakage; however, it is time‐consuming due to setup and sample preparation steps. DNA analysis, using assays such as Picogreen™ or spectrophotometric absorption devices such as NanoDrop™ offer another option to monitor cell lysis in fermentation. However, Picogreen™ assay requires a comparatively complicated protocol and is time‐consuming, and proteins can interfere in NanoDrop™ measurements as they are absorbed at the same wavelength as nucleic acids.^17^ In general, techniques for DNA analysis are susceptible to errors from losses due to degradation of DNA or from losses in sample preparation steps such as centrifugation.^17^ In situ probes using fluorescence and infrared spectroscopy are also available on the market and have been extensively studied.^19^, ^20^, ^21^, ^22^, ^23^, ^24^ They are capable of monitoring the chemical properties of fermentation broths such as biomass, glucose, and protein concentration. Although the available techniques can measure these analytes, fermentation broths are extremely complex and infrared spectroscopy can have problems with sensitivity when analysing molecules at low concentration, such as protein products or substrates such as glycerol or glucose.

Process analytical technologies (PAT) are highly desired in the biotechnology industry to aid bioprocess development, monitoring, and control,^25^, ^26^ and are particularly important for the implementation of quality‐by‐design initiatives.^27^ Analytical methods such as HPLC and flow cytometry present useful information for monitoring cell viability and product leakage; however, they are complex offline techniques. In contrast, offline viscosity measurements require no sample preparation, and analysis takes a fraction of the time. Additionally, viscosity measurements can be carried out at‐line and potentially online. The obtained real‐time data may be able to show the viability of high‐cell density *E. coli* cultures and detect the critical lysis point where product loss due to leakage occurs.

Rheology is defined as the study of the deformation and flow of matter, and can be divided into two subcategories; viscosity and viscoelasticity. In this study, we are predominantly concerned with viscosity measurements. Monitoring viscosity in fermentation has typically shown an increase in viscosity in relation to cell density in the exponential phase, followed by a subsequent immediate decrease in viscosity in the stationary phase.^28^, ^29^, ^30^ For mycelial fermentations, changes in cell morphology significantly affect broth viscosity.^17^ Fermentation broth viscosity is determined by both biomass concentration and liquor properties.^31^ Both protein concentration and DNA concentration will significantly influence the broth viscosity if present in high enough quantities. When cell lysis occurs, the intracellular content will leak into the broth and the viscosity will increase.

Viscosity monitoring during fermentation gained interest in the 70s and 80s, however, it lost momentum in the 90s, due to a lack of adequate technology to accurately monitor viscosity in complex systems, and its use predominantly focussed on monitoring biomass concentration. Viscosity measurements have been used previously to monitor cell concentration in filamentous fermentation broths, but with relatively poor results. A capillary‐type viscometer has been used to monitor the broth viscosity of *Hansenula polymorpha*, which was shown to increase nonlinearly with cell concentration.^32^ Using a rotational viscometer for *Saccharomyces cerevisiae* showed better results, although experiments were carried out using yeast cells resuspended in buffer solution.^33^ A vibrating rod has also been used to characterise the viscosity in xanthan production (*Xanthomonas campestris*) and yoghurt fermentation (*Streptococcus thermophilus* and *Lactobacillus bulgaricus*). This empirically based method has to be calibrated for each fermentation and the output signal transformed from voltage into arbitrary viscosity units.^34^ However, Vlahopoulou (1992) reported the use of dynamic oscillatory testing to investigate the effect of cell biomass and exo‐polymers produced by *Streptococcus theromphilus* and *Lactobacillus bulgaricus* strains during yoghurt fermentation.^35^, ^36^

The change in viscosity of *E. coli* suspensions has previously been observed whilst undergoing chemical (alkaline) lysis, however, this study monitored the change in viscosity over the course of the chemical reaction (10 minutes) to understand genomic DNA denaturation. Additionally, samples were taken at the beginning of the stationary phase where the cell density was low, there was no product involved, and the analysis method took 30–60 min.^37^ A recent study focussing on primary recovery (centrifugation and depth filtration) reported an increase in flow consistency index during fermentation, demonstrating that non‐Newtonian behavior increased during the fermentation,^38^ and indicated that monitoring this change in flow consistency index may be feasible for industrial fermentations to determine harvest time.

As the cell concentration increases, the viscosity also increases, however, the viscosity is not solely affected by cell concentration,^31^ and detailed characterization of viscosity changes during cell lysis in fermentation has not been carried out for the purpose of fermentation monitoring. We propose that monitoring viscosity may be an efficient way to indirectly infer cell lysis, to prevent product loss in fermentation and determine the optimal harvest time.

## Materials and Methods

### Strain

An *E. coli* w3110 strain (ATCC 27325) containing the plasmid pTTOD A33 IGS2, was kindly donated by UCB Pharma (Slough, UK), coding for a 46 kDa antibody fragment (Fab') utilising a *tac* promoter. All chemicals were provided by Sigma‐Aldrich (Dorset, UK) unless otherwise stated.

### Fermentation

High‐cell density fed‐batch fermentations were carried out using an autoclavable 7 L Applikon vessel (Applikon Biotechnology B.V., Schiedam, Holland), with a 5 L working volume. Cells were grown initially using complex LB broth, before being transferred to SM6Gc media, using a method previously described by Garcia‐Arrazola et al. (2005)^39^ and Li et al. (2012).^40^ On reaching an OD_600_ of around 200 (∼36 h postinoculation), a dissolved oxygen spike and pH spike indicated that the culture had utilized all of the glycerol carbon source in the media. At this point, isopropyl β‐D‐1‐thiogalactopyranoside (IPTG) (Generon, Maidenhead, UK) was added to a target bioreactor concentration of 0.03 g L^−1^ to induce Fab' expression, and 80% w/w glycerol solution was fed at a rate of 6.4 mL h^−1^. To control foaming, 1 mL of 100% polypropylene glycol (PPG) was added to the fermenter prior to inoculation, and as necessary thereafter up to a maximum of 2 mL total PPG. The fermentation was typically continued up to 60 h postinduction.

### Measurement of cell density

Optical density was measured at 600 nm using an Ultrospec 500 Pro spectrophotometer (Amersham Biosciences, Amersham, UK). Dry cell weight was measured by aliquoting 1 mL of culture into predried and preweighed 2 mL Eppendorf tubes, centrifuging, removing supernatant, and drying in an oven overnight at 100°C. Dry cell weight measurements followed the same trend as OD_600_ measurements, so are not shown in this article.

### Cell viability assays

Samples were sonicated to obtain data on the intracellular content. Sonication was carried out using a Soniprep 150 (MSE, London, UK), with 4 cycles each consisting of 10 seconds on, 10 seconds off, at an amplitude of 10 μm. A cytotoxicity assay, Cytotox‐96 (Promega, Madison, USA), was used to determine cell viability throughout the fermentation, based on lactate dehydrogenase (LDH) release. Quant‐iT Picogreen assay (Life Technologies, Warrington, UK) was used to determine double stranded DNA concentration in the fermentation broth.

### Fab' measurement

Fab' concentration in the supernatant and total Fab' concentration were analysed by HPLC (Agilent 1200, Agilent Technologies, CA) using a 1 mL protein G column (HiTrap, GE Healthcare, Uppsala, Sweden). To measure total Fab' concentration, sonication was carried out, followed by centrifugation. Bind and elute 20 mM phosphate buffers were used to process the samples, at pH 7.4 and pH 2.5, respectively. The concentration of eluted Fab' was measured by absorbance at 220 nm.

### Viscosity measurement

Rheological measurements were carried out with a Kinexus Lab + rheometer (Malvern Instruments, Malvern, UK), using 50 mm parallel plates at 25°C and a 300 μm gap size. Measurements were taken by controlling the applied shear rate over a range of 100–1000 s^−1^, and the apparent viscosity was determined by recording the viscosity value at a shear rate of 100 s^−1^.

### Capacitance measurements

The Aber Instruments (Aber Instruments, Aberystwyth, UK) Futura biomass probe (320 mm × 12 mm) was used in fermentations to obtain online, in situ measurements of viable cells. The probe was setup to measure in dual frequency mode, at 1.12 MHz and 15 MHz, which has been optimized for bacteria.

### Flow cytometry measurements: BP staining

Flow cytometry was performed using an Accuri C6 flow cytometer (BD Biosciences, CA), using bis‐oxonol (BOX) and propidium iodide (PI) stains. Analysis using this method has been described elsewhere.^41^ Briefly, fermentation samples were diluted to an OD_600_ of 1.0 AU and 10 µL combined with 990 µL of staining solution (50 µg/mL bis‐oxonol, 40 mM EDTA, 20 µg/mL propidium iodide, in PBS). Samples were stained for 8 min before analysis. Cells which remained unstained or stained with bis‐oxonol were counted to provide an overall measurement of viable cells. Flow cytometry measurements were verified by carrying out cell counts using colony forming units method.

### Scanning electron microscopy

Cells were re‐suspended for analysis in a primary fixative, 2% glutaraldehyde in 0.1 M sodium cacodylate buffer (pH 7.3), and left for 24 h at 3°C. The cells were then washed in a 0.1 M cacodylate buffer and fixed in a 1% osmium tetraoxide in 0.1 M cacodylate buffer at 3°C for 1.5 h. The cells were washed again in 0.1 M cacodylate buffer and washed with dH_2_O before dehydrating in a graded ethanol‐water series to 100% ethanol. The samples were then critical‐point dried using CO_2_, and mounted on aluminium stubs using sticky carbon taps. The samples were then coated with a thin layer of Au/Pd (∼2 nm thick) using a Gatan ion beam coater. The samples were viewed and imaged with a 7401 FEGSEM (Jeol, MA).

## Results and Discussion

### Rapid product loss and cell lysis in late stage fermentation

**Figure 1:**
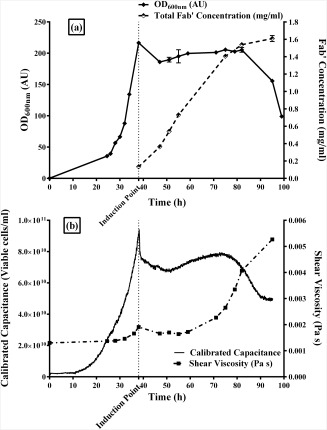
Characterization of cell lysis in an *E. coli* (Fab') fermentation. (a) Optical density at 600 nm (absorbance units (AU), in triplicate) and total Fab' concentration (mg/mL, induplicate). Induction time was at 38 h, using IPTG. (b) Capacitance profile (viable cells/mL, online continuous measurement) and shear viscosity (Pa s, single measurement, held at steady state for 10 s). Capacitance was calibrated offline with flow cytometry data (in triplicate).

The fermentation of *E. coli* has been carried out in fed‐batch mode in a 5 L working volume Applikon fermenter. As shown in Figure 1, the cells were seeded at an OD_600_ of 5 and grew in SM6Gc defined media. The exponential phase began at around 24 h and cells grew to a maximum dry cell weight of 48 g/L (corresponding to an optical density of around 200). Fab' expression was induced at 38 h when a dissolved oxygen spike was observed due to complete utilization of the carbon source (glycerol). The cells were induced with IPTG, which allowed rapid Fab' production in the stationary phase, reaching 1.6 g/L by the end of the fermentation. The stationary phase appeared to last until cell lysis was observed at 82 h (44 h postinduction, Figure 1a) from optical density measurements and 79 h (41 h postinduction, Figure 1b) from capacitance measurements of viable biomass. Throughout fermentations it was observed that foaming occurred during exponential phase. There were no specific observations relating to foaming in the stationary phase.

**Figure 2:**
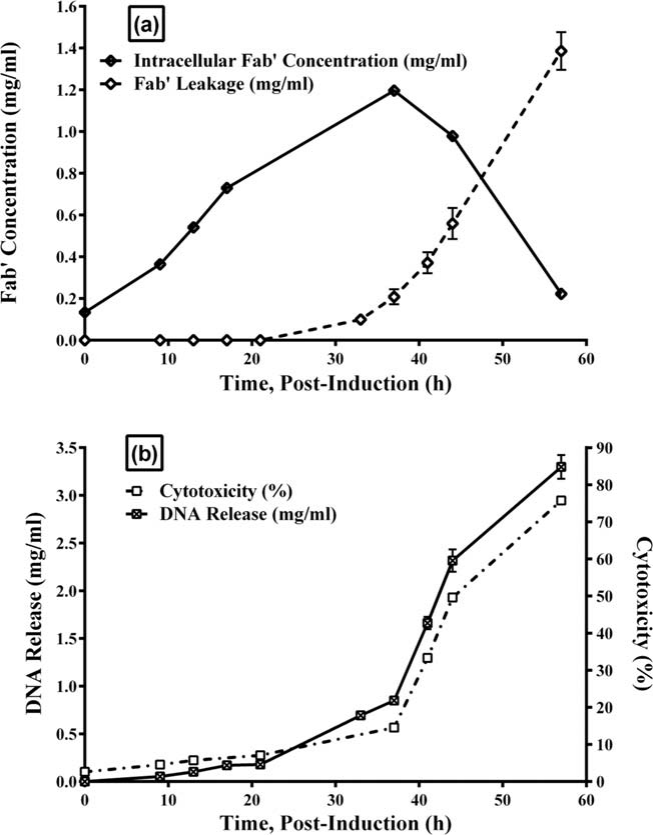
Analytical characterization of cell lysis in an *E. coli* (Fab’) fermentation. (a) Intracellular Fab' concentration and leakage of Fab' to extracellular space (postinduction, mg/mL, in duplicate). (b) Cytotoxicity (based on lactate dehydrogenase (LDH) release to extracellular space, %, in triplicate) and DNA release (postinduction, mg/mL, in triplicate).

As shown in Figure 2, however, maximum intracellular product concentration reached 1.2 g/L at 36 h postinduction, and 10% product leakage was observed at 33 h postinduction. If 36 h postinduction is selected as the harvesting time based on the highest intracellular titre, 25% of Fab' has already leaked out and the state of the cells deteriorates rapidly from this point on. For cell harvesting at manufacturing scale, such as centrifugation and/or microfiltration, the cells will have a high risk of lysis, leading to product loss due to the processing time and shear environment they are subject to. According to the optical density profile in Figure 1a, the first signs of cell lysis observed at 44 h postinduction corresponded to almost 40% product leakage, and for the capacitance data shown in Figure 1b signs of cell lysis at 41 h postinduction corresponded to 30% product leakage to the fermentation broth.

**Figure 3:**
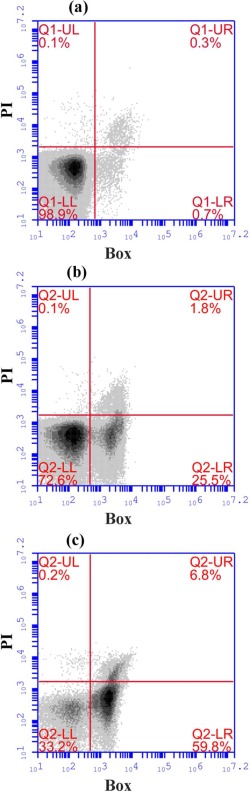
Flow cytometry plots for BOX (bis‐oxonol) and PI (propidium iodide) stains. For each plot, UL quadrant denotes dead cells and cell fragments, UR quadrant denotes PI stained cells (nonviable), LL quadrant denotes viable polarized cells, and LR quadrant denotes viable cells that have been stained by BOX (depolarized membrane). (a) Sample was taken in mid‐exponential phase, (b) sample was taken in mid‐stationary phase at the onset of cell lysis (36 h postinduction), and (c) sample was taken in late stationary/decay phase (57 h postinduction). Samples were measured in triplicate.

LDH is used as an indicator of cytotoxicity by detecting cell membrane integrity. Loss of membrane integrity implies loss of cell viability and product leakage. As shown in Figure 2b, significant DNA leakage and an increase in cytotoxicity occurred from 33 h postinduction, which correlated well with product leakage shown in Figure 2a. The flow cytometry plots in Figure 3 demonstrate this further; showing highly viable cells in exponential phase (99.6%, plot [a]), and in mid‐stationary phase (36 h postinduction) there are 98.1% viable cells (plot [b]). However, 25.5% of these cells have depolarized membrane channels (stained with bis‐oxonol). Technically, cells with depolarized membranes have the potential to recover if transferred to fresh media, however, will leak considerable quantities of product, DNA and other intracellular content if left in the fermenter. At 57 h postinduction, plot (c) in Figure 3 shows there are only 93% viable cells (33.2% polarized, 59.8% depolarized), with 6.8% nonviable cells (stained with propidium iodide). However, at this point, almost 90% of Fab' product had been lost to the fermentation broth. Therefore, although cells with depolarized membrane channels are still technically “viable,” in terms of fermentation, it can be said that they have lost the ability to produce and retain product in the periplasm. Traditionally, the definitions of lysis and viability are that if cells are leaking DNA, lysis is occurring. If cells are leaking product, that is, have a depolarized, nonintact membrane, they are nonviable. Figures 2 and 3 demonstrate that lysis, loss of viability and product loss occur simultaneously in fermentation.

**Figure 4:**
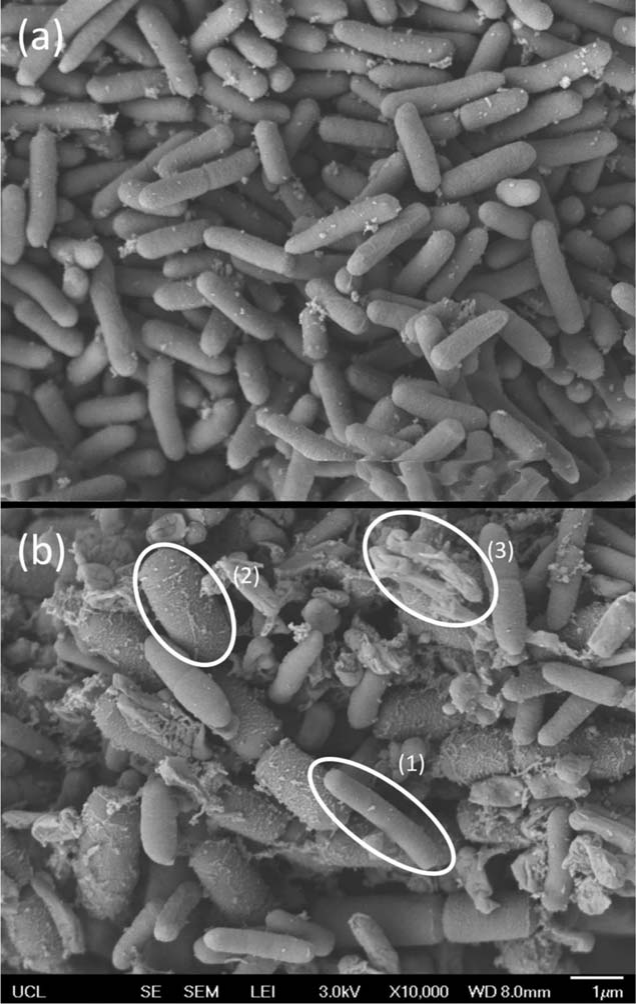
Scanning Electron Microscopy (SEM) images at x10,000 magnification. (a) SEM image of an *E. coli* fermentation sample showing predominantly viable cells in early stationary phase. (b) SEM image of an *E. coli* fermentation sample in late stationary/decay phase, showing; (1) healthy cells, (2) swollen cells, and (3) shells of lysed cells.

Scanning electron microscopy (SEM) images were taken of *E. coli* throughout a fermentation to provide qualitative observation, a selection of which are shown in Figure 4. Figure 4a shows highly viable cells, with largely intact cell membranes in early stage postinduction culture. Figure 4b shows three things in late stage postinduction culture; some healthy viable cells, swollen cells and empty shells of lysed cells. This suggests that the over‐expression and subsequent build‐up of product within the periplasm (capacity of Fab' in the periplasm is limited to 6%) has a significant stress on the metabolism of the cells, leading to a loss of the cell's ability to maintain their osmotic balance in late‐stage fermentation, seen by the swelling and bursting of cells.

Although optical density and capacitance measurements indicate the onset of cell lysis at a much later time‐point, HPLC measurements show 10% product leakage at 33 h postinduction and a subsequent rapid increase in product loss. Apart from the OD_600_ and capacitance data, we can see that flow cytometry, DNA leakage and cytotoxicity data all agree with the HPLC data, and signs of cell lysis are evident from 33 h postinduction.

### Viscosity monitoring as an indicator of cell lysis

**Figure 5:**
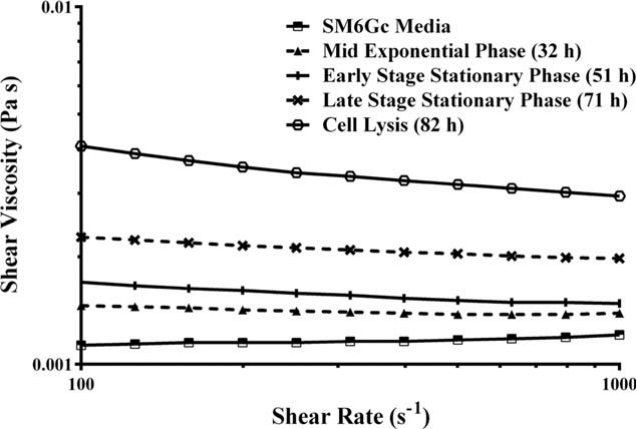
Viscometry flow curves of *E. coli* cell broth at various times during the fermentation, over a shear rate range 100–1000 s^−1^. Induction time was at 38 h, using IPTG. The viscometry measurements were carried out at 25°C using 50 mm parallel plates. An increase in shear thinning behavior is evident as the fermentation proceeded (flow behavior index was greater than 0.95 for all samples). Single viscometry measurements were recorded at each shear rate, held at steady state for 10 s.

Shear thinning behavior is often observed at low shear rates in fermentation broths with high cell densities that have been partially or wholly disrupted to release intracellular content without degrading high molecular weight nucleic acids; for example, after homogenization using low pressures and a minimum number of passes,^40^ or during cell lysis in fermentation. This shear thinning behavior is thought to be caused by structural interactions between high molecular weight nucleic acids, cells, and cell debris in the fermentation broth. As shown in Figure 2b, significant quantities of high molecular weight DNA (and other intracellular content) were released to the broth as the fermentation progressed, which contributed to an increase in shear thinning behavior, shown in Figure 5. Flow behavior index is measured by the linearity of the flow curve. A Newtonian fluid has a flow behavior index of 1. As the flow behavior index decreases below 1, the material displays increasing shear thinning behavior. However, the flow behavior index remained above 0.95 for all flow curves shown in Figure 5, displaying only mild shear thinning behavior. This shows that there is only a slight structure in the samples, as low cell concentrations are present in fermentation broths in comparison to homogenization feeds (which typically have significant structure).

Viscosity measurements were recorded for each shear rate when steady state values were maintained over a 10 second period. A shear rate of 100 s^−1^ was chosen as the characteristic parameter for obtaining viscosity values because at lower shear rates the measurement will pick up more of the polymer interactions (e.g., DNA) that are thought to cause an increase in viscosity during cell lysis. The viscosity profile shown in Figure 1b increased correspondingly to the increase in cell density during exponential phase up to 38 h, at which point a relatively flat viscosity profile was observed until 71 h (33 h postinduction), where the viscosity was seen to rapidly increase. Although the cell density increase in exponential phase was significant, the resulting increase in viscosity was relatively small. As cell lysis progressed, there was a much larger increase in viscosity.

The postinduction viscosity profile closely follows the cell lysis trends observed through HPLC, flow cytometry, DNA release, and cytotoxicity; however, the viscosity increase can be detected more rapidly than the other analytical methods presented. In comparison to HPLC, the up‐front capital cost is much lower for the rheometer, the maintenance costs and cleaning downtime are minimal, and no reagents are needed for the rheometer.

**Figure 6:**
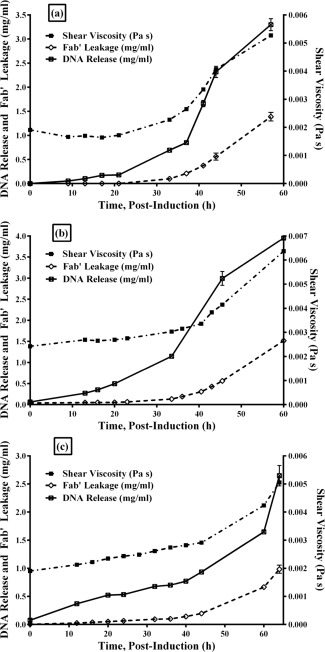
Effect of product leakage (mg/mL, measured in duplicate) and DNA release (mg/mL, measured in triplicate) on viscosity increase (Pa s, single measurement, held at steady state for 10 s) in postinduction culture for three fermentation runs.

Figure 6 shows three different fermentation runs and how they correlate with viscosity; 10% leakage of product to the fermentation broth corresponded to a 25% (+/‐ 5%) increase in broth viscosity (with induction‐point viscosity as a reference, to negate differences in cell density between batches). It is straightforward to characterize the viscosity profile of a fermentation system in this way, and correlate this to product leakage and cell lysis. Using the results presented in this article to exemplify a practical application of this technique; based on measurement variation, an increase in viscosity above 20% (postinduction) may be an appropriate time to end the batch, taking into consideration the downstream processing time and operating environment the cells are subject to. To characterize a different fermentation system, one should start by assessing the intracellular product profile of their system, take into account scale and downstream processing requirements, and then determine the optimum harvest point. The harvest point can be correlated to the viscosity profile, and viscosity monitoring can then be used to rapidly determine the harvest point in subsequent fermentations. This shows that viscosity monitoring may be used as an indicator of cell lysis in postinduction cell cultures, and used to aid process development and process operation at manufacturing scale due to its ability to rapidly monitor the physical properties of cell cultures to prevent product loss and avoid further complications downstream.

## Conclusions and Future Work

It has been shown that viscosity monitoring can detect cell lysis and product leakage in postinduction cell cultures. Several common techniques for monitoring cell lysis have been presented and compared, and viscosity monitoring was shown to perform better than OD_600_ measurements and online capacitance probes, but has equivalent performance to HPLC, flow cytometry, cytotoxicity assays, and DNA quantification. However, monitoring viscosity at‐line can provide information on lysis in a much shorter time period than the other methods presented; data can be obtained in under 2 minutes, which enables the operator to make rapid decisions about cell harvesting.

This study has also demonstrated that significant value can be found in using viscosity to monitor the physical properties of cell cultures to infer cell lysis, and could be used in conjunction with infrared monitoring to give a more comprehensive picture of the bioprocess by monitoring both the physical and chemical properties of the broth.

We conclude that a combination of DNA, protein and other intracellular content may cause the increase in viscosity in postinduction cell culture. However, the picture is not yet clear; complex interactions between biomass and their polymers may be present in the composite system.^14^, ^35^, ^36^ Therefore, further studies are required to determine exactly what contribution each of these components makes to the increase in viscosity, and if there are important interactions between cells, cell debris, and other intracellular material. Additionally, this study has demonstrated that significant value can be found in developing an online, in situ viscosity probe for fermentation monitoring.
